# Examination of Resident Physician Quality Improvement/Patient Safety Project Confidence Levels from Multiple Programs

**DOI:** 10.51894/001c.5097

**Published:** 2016-10-24

**Authors:** Carolyn McGrail, Josie Urban, Brandy Church, William D. Corser

**Affiliations:** 1 Authority Health, Detroit, MI; 2 Michigan State University College of Osteopathic Medicine, Statewide Campus System

**Keywords:** project confidence, scholarly activity, patient safety, quality improvement

## Abstract

**CONTEXT:**

It is now increasingly recognized that physicians should be engaged in quality improvement/patient safety (QIPS) activities to make their patient care systems perform more reliably and safely. In order to ensure that our nation’s physicians embed this aspect of practice into their work, there also is a growing expectation for effective integration of QIPS training into graduate medical education. This exploratory pilot study was conducted to identify how residents’ personal and residency program characteristics might be related to their perceived confidence to develop and conduct prospective QIPS projects.

**METHODS:**

A total non-probability convenience sample of 43 DO resident physicians from five residency programs (Family Medicine, Internal Medicine, Obstetrics and Gynecology, Pediatrics, and Psychiatry) at Authority Health were surveyed from 09/28/2015 to 01/06/2016 using online Survey Monkey software. A 38-item survey asked residents about their personal and residency program characteristics, as well as their current overall perceived confidence to develop and conduct QIPS projects.

**RESULTS:**

Two model terms that proved non-significant during analyses were residents’ age category and year in residency training. In the final stepwise multinomial regression model, however, three covariates including: a) sex (p=0.045), b) being in a primary care residency program (p=0.038) and c) having had prior QIPS project experience (p=0.049) were each found to be statistically significant predictors of respondents’ perceived comfort level categories. Male residents and those who were in a primary care residency program (i.e., Family Medicine, Internal Medicine or Pediatrics), and/or reported having had prior QIPS project experience, reported significantly higher confidence levels.

**CONCLUSIONS:**

Somewhat similar to earlier studies, these results suggest the need to incorporate QIPS education for resident trainees across the nation. Ideally, the findings from larger resident studies will enable GME leaders to develop and deliver evidence-based QIPS curricula that are better oriented to resident physicians’ personal characteristics and preferences.

## INTRODUCTION

National concerns regarding an improved awareness of patient safety and quality of care within the US health care system have driven expectations for a minimal competence level in quality improvement/patient safety (QIPS) from contemporary physicians. It has been increasingly recognized that physicians should be engaged in QIPS activities to make the systems through which they provide patient care perform more safely and reliably.^1,2^

In order to ensure that our future physicians embed this aspect of practice into their work, there also is a growing mandate for the effective integration of QIPS training into graduate medical education (GME).^3^ A broader term used within *Next Accreditation System* documents has been *scholarly activity,* referring to *both* healthcare systems-oriented QIPS projects and formal controlled research studies.^4,5^

The overall rationale for such integration of QIPS content into GME curricula is a belief that when residents develop and apply project design/conduction skills, they will continue to participate in such activities once they enter post-residency practice.^3,5^ In this respect, the Accreditation Council for Graduate Medical Education (ACGME) 2013 Common Program Requirements establish that ‘’residents must demonstrate the ability to investigate and evaluate their care of patients, to appraise and assimilate scientific evidence, and to continuously improve patient care based on constant self-evaluation and life-long learning.’’ ^6(p11)^

It is now also considered mandatory by the ACGME that residents learn to ’’systematically analyze practice using QI methods and implement changes with the goal of practice improvement.”^6^ An ongoing challenge for GME leaders and faculty, however, has been the lack of specific curricular frameworks to help residents plan out QIPS projects in diverse community-based residency program settings.^7–9^

The importance of establishing coordinated scholarly activity (SA) project expectations and timelines for residency programs has been suggested by a growing number of GME experts.^3,10^ During recent years it also has been proposed that coordinated efforts by GME officials from multiple residency programs and systems may enable them to more efficiently share resources, resulting in more QIPS projects and products.^3,11,12^

In terms of preparing residents for QIPS project experiences, some authors have reported achieving increased momentum from the establishment of cross-program project teams with shared project interests.^13–17^ Other GME groups have specifically implemented residency program timelines during which earlier-year residents develop specialty-related project conduction skills to lead their own later-year projects.^3,13,18–20^

Similar to what the authors have seen in several affiliated healthcare systems, councils or committees have been established in some settings to coordinate resident or faculty-resident project planning.^17,21,22^ Some GME publications have described *rotations* for residents to work individually with healthcare system QIPS department personnel to generate their own subsequent QIPS project ideas.^23–26^

Since they lacked an institution-wide QIPS curriculum, GME faculty (CM, JU) at Detroit Wayne County Health Authority (Authority Health) in Detroit, MI concluded that it was first necessary to determine their residents’ baseline QIPS project-related confidence levels. The development of this study started as an 18-month *Teach for Quality* GME project coordinated by the third author (BC). The Association of American Medical College’s *Teach for Quality* program was designed in 2013 to equip clinical faculty to lead, design, and evaluate effective QIPS projects.^27^ The authors (BC, WC) from the Statewide Campus System ^28^ (SCS) of the Michigan State University College of Osteopathic Medicine adapted the training program to support a cohort of GME faculty learners through a consortium model of QIPS coaching supports and materials. The overall results of this QIPS-oriented curricular series of 13 developed projects have been reported in another paper available from the authors.^22^

## PROJECT PURPOSE

This exploratory pilot study was conducted to identify how residents’ personal and residency program characteristics, demographics, and previous QIPS project experience might influence residents’ perceived confidence levels to develop and conduct prospective QIPS projects. The authors’ intent is that the results from this project inform future work and integration of QIPS content and mentoring into residents’ training at Authority Health and at other individual residency programs. The overall null hypothesis of the study was that there would be no statistically significant differences between subgroup QIPS confidence level responses based on any residents’ personal or residency program characteristics.

## METHOD

After obtaining IRB approval, a total non-probability convenience sample of 55 resident physicians from five residency programs (Family Medicine, Internal Medicine, Obstetrics and Gynecology, Pediatrics, and Psychiatry) at Authority Health were surveyed by the first two authors (CM, JU) from 09/28/2015 to 01/06/2016 using online Survey Monkey software.^29^ This software allowed for anonymous survey submissions and easy extraction of raw response data. Prospective respondents were first emailed a survey link through their residency program emails, and later reminded to consider participating in the study through additional emails and by the first two authors (CM and JU) at agency/program staff meetings.

The complete 38-item project survey included a QIPS project confidence portion derived from the 31-item *Quality Improvement Confidence Instrument* (QICI) developed and psychometrically validated by Hess et al. in 2013.^30,31^ After respondents were asked seven questions about their personal and residency program characteristics, they were presented with 31 questions about their perceived confidence in their ability to develop and conduct QIPS projects. Each of the 31 items used a five-point scale ranging from *Not At All Confident* to *Very Confident*. The six subscales of the QICI included: 1. D*escribing the Issue*, 2. *Building a* *Team*, 3. *Defining the Problem*, 4. *Choosing a Target*, 5. *Testing the Change*, and 6. *Extending Improvement Efforts*.^30,31^ Respondents also were asked for suggestions of QIPS topics they might be interested in learning more about.

Following completion of data collection, data cleaning was undertaken using SPSS version 22 analytic software^32^ to transform nominal response data into numeric form and collapse some diverse variables into categories. A series of descriptive statistics were generated with cross-tabulations, and correlations were calculated among five key selected respondent characteristics the authors hypothesized might influence respondents’ overall QIPS project confidence levels (age, gender, residency year, type of program, previous QIPS experience). Participants also were asked whether they had obtained any prior experience in QIPS-type activities.

Cleaned study data were analyzed using a series of forward stepwise multinomial logistic regression modeling (MLM) procedures.^33^ These procedures were used to determine if any statistically significant associations existed between five resident characteristics with adequate frequencies and their respective QIPS project confidence levels. These types of analytic procedures are especially suited for smaller samples in which prospective cell frequencies (e.g., Male Pediatrics or Female Psychiatry resident respondents) are likely to be lower and not normally distributed.^33^

## RESULTS

*Descriptive Statistics*: Of the 55 respondents who started the online survey, a total of 43 (78% of total respondents) completed 95% or more of the entire survey. Twelve (22%) respondents apparently either failed to note that the survey was comprised of additional survey screens or decided they were uninterested in completing the survey. The final analytic sample used for most analyses therefore was comprised of the 43 respondents who had completed most items on the entire survey.

The authors began by comparing the reported personal characteristics of the 12 respondents who failed to complete the QICI section of the survey to the 43 respondents who completed the entire survey tool. Using these fields completed by the totality of the respondent sample, a series of cross-tabulation tests comparing the two sample subgroups failed to show any statistically significant differences between partial-survey and full-survey respondents.

In the final analytic study sample (n = 43), 26 (60%) respondents were female, with respondents’ reported age category split fairly evenly between the *20 to 30 years of age* and the *31 years and older* categories. Respondents also were approximately uniformly divided across *First-year* through *Third or Fourth-year* residency program year categories.

In order to divide the sample into comparison groups based on type of training program, the authors created a *Primary Care* category (Family Medicine and Pediatrics) that comprised 54% of the analytic sample. The *Non-Primary Care* category was comprised of Internal Medicine, Obstetrics/Gynecology and Psychiatry respondents. A total of 18 respondents (42%) indicated that they had participated in some sort of prior QIPS project experience (see Table 1).

**Table 1.** Descriptive Characteristics of Sample (n = 43)

**Age Category**	**N (%)**
20 to 30 years old	23 (53.5)
31 years or older	20 (46.5)
**Gender**	
Male	17 (39.5)
Female	26 (60.4)
**Residency Program Year**	
First Year	11 (25.6)
Second Year	16 (37.2)
Third or Fourth Year	16 (37.2)
**Type of Residency Program**	
Family Medicine	11 (25.6)
Internal Medicine	8 (18.6)
OB/GYN	3 (7.0)
Pediatrics	12 (27.9)
Psychiatry	9 (20.9)
**Primary Care (Family Medicine and Pediatrics)**	
Yes	23 (53.5)
No	20 (46.5)
**Prior QIPS Project Experience**	
Yes	18 (41.9)
No	25 (58.1)

*Respondents’ Overall QIPS Confidence Levels:* Before respondents answered the 31 question QICI survey, they were asked a summary item concerning their overall level of confidence in designing and implementing a QIPS project. Responses on the five-point scale were grouped into three levels: a) *Not Comfortable* 21 respondents (49%), b) *Somewhat Comfortable* 16 (37%) and c) *Pretty Comfortable* or *Very Comfortable*” 6 (14%).

An initial forward stepwise MLM was conducted comparing the major resident characteristics to prospective QIPS project comfort levels. A two-tailed p-value=0.05 was selected to designate statistical significance. Each model term was entered individually in a stepwise manner to gauge their significance for the three overall comfort level response categories. Those variables initially found to exert non-significant degrees of significance (p-value of greater than 0.100) were removed from the later final MLM model.

**Figure 1.**
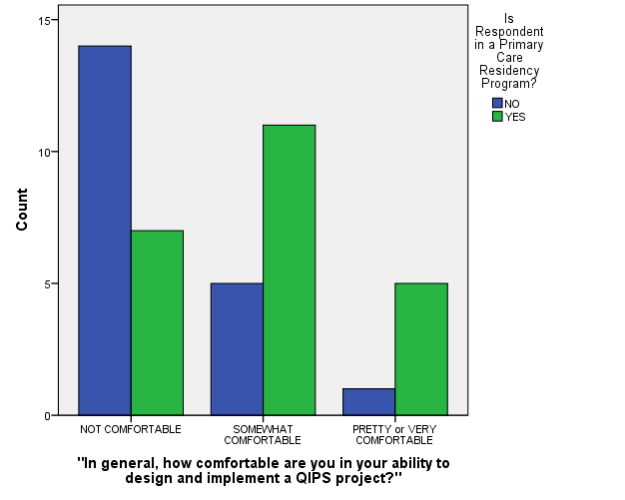
“In general, how comfortable are you in your ability to design and implement a QIPS project?”** (stratified by Primary Care versus Non-Primary Care Program)

**Figure 2.**
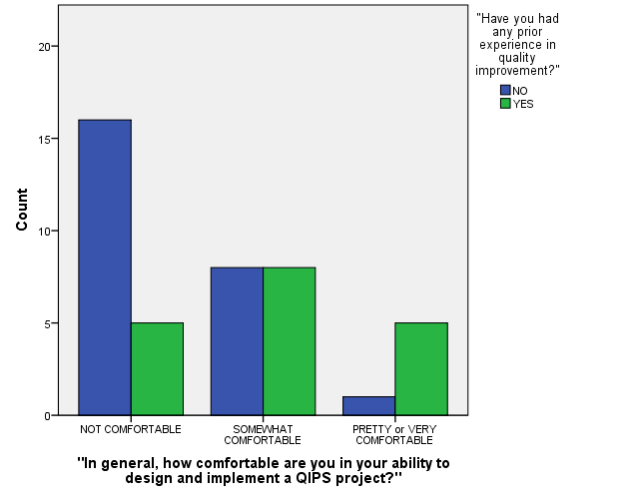
“In general, how comfortable are you in your ability to design and implement a QIPS project?”** (stratified by whether or not Respondent reported Prior QIPS Project Experience)

Two MLM model terms that were not significant in the initial model were *Age Category* (confined to two categories since all respondents were either in their twenties or thirties) and *Year of Residency Program* (three categories). In the final regression model, however, three factors were each found to be statistically significant predictors of respondents’ reported confidence level categories (see Figures 1-2 and Table 2 for final model test statistics) These were: a) Gender (p=0.045), b) being in a Primary Care residency program (p=0.038) and c) having had prior reported QIPS project experience (p=0.049). In summary, males, those residents who were in a Primary Care program, and/or those who reported having had prior QIPS project experiences reported significantly higher levels of QIPS project confidence.

**Table 2.** Significant Predictors of Respondent Comfort Levels Designing and Conducting a QIPS Project (at time of survey) (N = 43) *

	Model Fitting Criteria	Likelihood Ratio Tests	
	**-2 Log Likelihood of Reduced Model**	**Chi Square**	**P-Value**
**Constant**	53.365		
**Gender (reference group Males)**	59.581	6.216	**0.045**
**Primary Care (ref. group Non-Primary Care )**	60.377	7.012	**0.030**
**“Do you have any Prior QIPS Project Experience?” (ref. group “No”)**	61.479	8.114	**0.049**

**Bold**: P-values significant at Alpha of *p* < 0.05.

* Forward Stepwise Multinomial Logistic Regression Models

The *final model fitting* test statistic generated by the analytic software was 0.05, indicating that the set of selected model terms used performed significantly better than an *intercept-only* model. The McFadden *Pseudo R-Square* test statistic generated by the software, however, was a fairly modest 0.231. This indicated the three surviving model terms (listed in prior paragraph) explained a relatively small proportion of sample respondents’ confidence levels.^33^ In other words, other unmeasured resident factors likely may have influenced how this sample of residents responded to this composite confidence item.

As depicted in Table 3, the distribution of the composite QICI and six subscale scores was quite considerable, generally ranging four to fivefold. Using a series of different stepwise linear regression models, however, none of the three resident characteristics that survived in the final MRM models came up as statistically significant influences for either composite or subscale confidence scores. This finding may be conservatively interpreted as an indication that respondents’ confidence scores may have been affected by interactive (i.e., combined factor), and/or non-linear unmeasured relationships within this sample.

**Table 3.** Quality Improvement Confidence Instrument Composite and Subscale Scores *

	**N**	**Minimum Response Received**	**Maximum Response Received**	**Mean**	**SD**
**Composite QICI Confidence Score**					
(possible range from 31 to 155)	43	42	145	95.02	26.769
***Describe the Issue* Subscale Score**					
(4 items) (possible range from 5 to 20)	43	5	20	12.35	3.841
***Build a Team* Subscale Score**					
(4 items) (possible range from 5 to 20)	43	5	20	13.42	4.193
***Define the Problem* Subscale Score**					
(5 items) (possible range from 5 to 25)	43	5	25	16.26	4.562
***Choose a Target* Subscale Score**					
(2 items) (possible range from 2 to 10)	43	2	10	5.79	2.166
***Test The Change* Subscale Score**					
(7 items) (possible range from 7 to 35)	43	7	35	18.49	7.756
***Extend Improvement Efforts* Subscale Score**					
(9 items) (possible range from 9 to 54)	43	12	44	28.72	8.09

* Five-point scale ranging from **Not At All Confident* to *Very Confident**.

Eight residents responded to the open-ended “topics of interest” survey item, citing categorized areas such as: a) *basic training regarding QIPS project conduction* (n = 5), b) *patient wait time* (n =1), or c) *delivering more cost effective healthcare* (n = 2).

## LIMITATIONS

These study results have two major limitations that the authors would like to acknowledge: a) the smaller single-setting convenience sample and b) potential methodological problems of breaking residency programs into Primary Care and Non-Primary Care categories. If the study sample had been larger and derived from multiple settings, other sample subgroup differences may have been observed. It also should be acknowledged that the authors’ decision to categorize certain respondents into a Primary Care category and others into a separate non-Primary Care category was somewhat forced by the distribution of residents across Authority Health’s training programs. Obviously, none of these survey items measured respondents’ actual competency to design or conduct QIPS projects.

## CONCLUSIONS

The authors could confidently reject their null hypothesis since the results demonstrated such variation in respondents’ QIPS project confidence levels. Somewhat similar to earlier studies, significantly higher QIPS project confidence levels were obtained from males,^30^ primary care residents,^34^ and those with some form of prior QIPS project experience.^35^ Due to the wide variety of medical skills that many primary care physicians may need to practice, it was not unexpected that primary care respondents would report higher baseline QIPS confidence participating in a new process than their counterparts.

These results suggest the need across residency programs for systematic QIPS education to enable physician trainees to gain confidence to incorporate QIPS project principles into their medical practices. Although the authors’ level of statistical power to detect interactive or smaller subgroup differences was certainly limited in this study, the complex variations found in even this smaller sample may have implications for GME faculty. These results could suggest what QIPS content topics may be more important for suitable residency program curricula. Larger scale follow-up/longitudinal studies are certainly required to generate more generalizable results for GME settings across the country. Ideally, future studies will enable GME leaders to develop and deliver effective QIPS curricula based on resident physicians’ characteristics and preferences.

### Conflict of Interest

The authors declare no conflict of interest.
